# Subgingival microbiome in patients with healthy and ailing dental implants

**DOI:** 10.1038/srep10948

**Published:** 2015-06-16

**Authors:** Hui Zheng, Lixin Xu, Zicheng Wang, Lianshuo Li, Jieni Zhang, Qian Zhang, Ting Chen, Jiuxiang Lin, Feng Chen

**Affiliations:** 1 Department of Orthodontics, Peking University School and Hospital of Stomatology, Beijing 100081, China; 2 The Third Dental Center, Peking University School and Hospital of Stomatology, Beijing 100011, China; 3 Bioinformatics Division, TNLIST and Department of Automation, Tsinghua University, Beijing 100084, China; 4 Central Laboratory, Peking University School and Hospital of Stomatology, Beijing 100081, China; 5 Bioinformatics Division, TNLIST and Department of Computer Science and Technology, Tsinghua University, Beijing 100084, China; 6 Program in Computational Biology and Bioinformatics, University of Southern California, Los Angeles, CA 90089, USA

## Abstract

Dental implants are commonly used to replace missing teeth. However, the dysbiotic polymicrobial communities of peri-implant sites are responsible for peri-implant diseases, such as peri-implant mucositis and peri-implantitis. In this study, we analyzed the microbial characteristics of oral plaque from peri-implant pockets or sulci of healthy implants (n = 10), peri-implant mucositis (n = 8) and peri-implantitis (n = 6) sites using pyrosequencing of the 16S rRNA gene. An increase in microbial diversity was observed in subgingival sites of ailing implants, compared with healthy implants. Microbial co-occurrence analysis revealed that periodontal pathogens, such as *Porphyromonas gingivalis*, *Tannerella forsythia*, and *Prevotella intermedia*, were clustered into modules in the peri-implant mucositis network. Putative pathogens associated with peri-implantitis were present at a moderate relative abundance in peri-implant mucositis, suggesting that peri-implant mucositis an important early transitional phase during the development of peri-implantitis. Furthermore, the relative abundance of *Eubacterium* was increased at peri-implantitis locations, and co-occurrence analysis revealed that *Eubacterium minutum* was correlated with *Prevotella intermedia* in peri-implantitis sites, which suggests the association of *Eubacterium* with peri-implantitis. This study indicates that periodontal pathogens may play important roles in the shifting of healthy implant status to peri-implant disease.

Implants have revolutionized dental rehabilitation, prosthetic dentistry, and maxillary reconstruction^1^,^2^. Marketing estimates show that over 2 million dental implants were inserted annually in the United States at the turn of the millennium^3^. Although dental implants survive well, infections at peri-implant sites have been widely reported^4^,^5^,^6^. Peri-implant diseases present in two forms: peri-implant mucositis (PM) and peri-implantitis (PI). In PM, inflammation is confined to the soft tissues surrounding a dental implant, with no sign of any loss of supporting bone after the initial bone remodeling that takes place during healing^5^. PI is characterized by inflammation around the implant, involving both soft tissues and a progressive loss of supporting bone to an extent greater than occurs upon biological remodeling, and may eventually lead to loss of the implant (implant failure)^7^. Peri-implant diseases have become emerging problems as the number of implants placed increases. The prevalence of mucositis is ~80% in implant patients and ~50% in the implants per se, whereas peri-implantitis has been diagnosed in 28–56% of implant patients and 12–43% of implants^8^,^9^. Bacteria colonize the peri-implant crevice soon after implant placement to establish polymicrobial communities^9^,^10^, and the failure of dental implants is commonly ascribed to inflammation of the supporting bone and related soft tissues caused by microbiota in peri-implant biofilms^11^,^12^.

PM and PI correspond in basic terms to gingivitis and periodontitis. Persistent gingivitis may lead to chronic periodontitis in susceptible individuals^13^. From the viewpoint of microbial ecology, red and orange complexes are more prevalent and more numerous in the lesions of established gingivitis, and this is even more apparent in periodontitis^14^. The microbial compositions of gingivitis have been compared with those of PM^15^, and those of periodontitis with PI^16^,^17^,^18^. However, PM, regarded as the precursor of PI, has seldom been investigated separately, and the relationships between the microbial communities of PM and PI remain unclear.

Traditionally, studies on the pathogenesis of peri-implant microbiota have analyzed individual bacterial species in complex microbial communities. More recent work has shown that peri-implant diseases may be polymicrobial in etiology, caused by a shift in the microbial community, rather than a single pathogen^16^. Previous studies, using culture-based methods, 16S rRNA gene PCR, or DNA-DNA hybridization techniques, commonly addressed roles played by individual bacterial species and afforded limited information on the overall diversity of the peri-implant environment. Sequencing of 16S ribosomal genes has yielded deeper insights into the composition of the oral microbiome in health and disease, creating a paradigm shift in our understanding of such microbial communities^19^. Pyrosequencing of PCR-amplified 16S rRNA is a next-generation sequencing method that simultaneously generates thousands of sequences from individual samples. Such an unprecedented amount of information allows comprehensive examination of a taxonomically heterogeneous community and has revealed ever-greater levels of microbial diversity^20^,^21^. A recent study on peri-implant bacterial communities using 16S pyrosequencing revealed that the microbial profile of healthy implants was significantly more diverse than that of PI sites^16^. However, when the prevalence of individual species was evaluated using DNA-DNA hybridization methods, Renvert S. *et al.*^6^ found no difference in microbial diversity between PI and healthy sites, whereas others detected fewer species in healthy sites compared to PI sites^22^,^23^.

In the present study, we analyzed subgingival plaque samples from healthy implants, PM and PI, using the 16S rRNA pyrosequencing method. This study was performed to compare the differences of the microbial communities of healthy implants, PM and PI, aiming to reveal the potential pathogens associated with peri-implant diseases.

## Methods

### Subject recruitment

Ten individuals with healthy peri-implant sites (n = 10), eight cases with PM (n = 8), and six cases with PI (n = 6), participated in the study. The project was approved by the Peking University Biomedical Ethics Committee (Beijing, China). Subjects gave written informed consent with the approval of the Ethics Committee of the Peking University School and Hospital of Stomatology. The methods were carried out in accordance with the approved guidelines. All patients had received dental implants in our hospital using the Straumann Dental Implant System (Straumann, California, USA).

### Diagnosis and sample collection

The diagnostic criteria for peri-implant diseases were in accordance with the recognized definitions of PM and PI^8^. Plaque samples were collected from peri-implant sulci or pockets, at the maximum possible probing depth, using a sterile periodontal probe.

### Microbial DNA extraction, 16S rRNA gene library preparation, and pyrosequencing

DNA from plaque samples was extracted and the v1–v3 hypervariable regions of bacterial 16S ribosomal RNA genes were amplified via PCR. The libraries were pyrosequenced on a 454-GS-FLX sequencing platform (454 Life Sciences, Branford, USA) at the BGI Institute (BGI Institute, Shenzhen, China). These sequence data have been submitted to the Short Reads Archive (Accession number SRP043555).

### 16S data processing and statistical analysis

The raw sequencing data were analyzed using (principally) the pipeline tools MOTHUR^24^ and QIIME^25^, as described in supplementary methods. Student’s *t*-test was used to compare alpha and beta diversities. Differences in the relative abundances of taxa in healthy implant, PM, and PI samples were analyzed using the Wilcoxon rank-sum test. Differences in prevalence were compared using Fisher’s exact test. P values <0.05 were considered to indicate statistical significance. P values have not been corrected for multiple comparisons. We calculated the Pearson correlation coefficients (PCC) for each pair of operational taxonomic units (OTUs) and used the permutation test to compute the statistical significance of the PCC value. Edges were set between pairs of OTUs for which the PCC was significant (P < 0.01).

### Quantification of bacterial loads of the *Eubacterium brachy* subgroup

Bacterial loads of members of the *Eubacterium brachy* subgroup were determined via real-time PCR using modified genus-specific primers^26^.

Detailed methods were provided as Supplementary data.

## Results

### Peri-implant diseases were associated with increased microbial diversity

**Figure 1:**
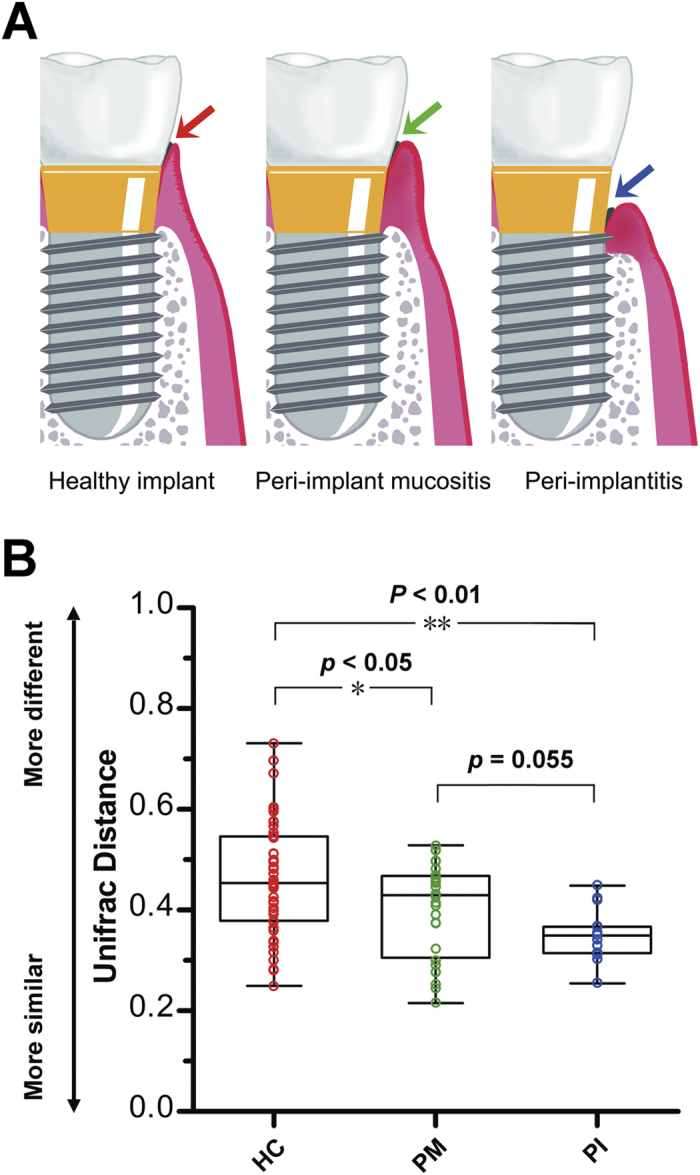
Sample collection and microbial community variation within groups. (**A**) A diagrammatic representation of our sample collection procedure. Plaque from healthy implant, peri-implant mucositis, and peri-implantitis sites was sampled from the deepest pockets or sulci. (**B**) The average weighted UniFrac distance values (the beta diversities) of healthy implant (HC), peri-implant mucositis (PM), and peri-implantitis (PI) sites. Healthy implant sites tended to host diverse bacterial communities, whereas peri-implantitis sites showed the greatest similarity in microbial communities. *P < 0.05, **P < 0.01 by two-tailed *t*-test.

Schematic diagrams of healthy implant, PM and PI are shown in Fig. 1A. The demographic and clinical parameters of all subjects are shown in Table 1. In total, 424,579 final reads were generated after processing, with a mean of 17,692 ± 6,236 (range 9,720–38,763) per sample. We finally detected 15,766 OTUs, with 311–1,028 OTUs in individual specimens, using a 97% similarity cutoff (for details please see Table S1).

**Figure 2:**
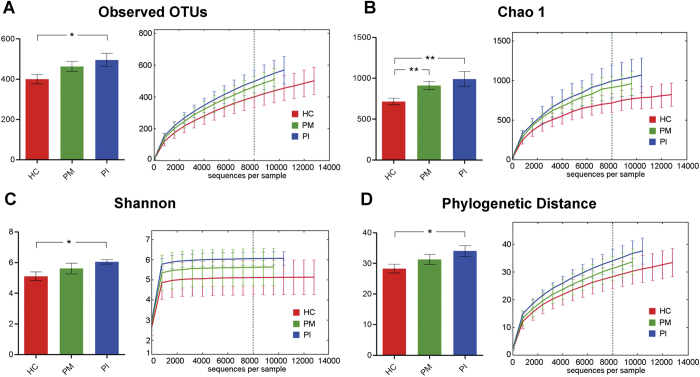
Calculation of alpha diversity values for comparison of the total microbial diversity of healthy implant (HC), peri-implant mucositis (PM), and peri-implantitis (PI) sites. Alpha diversity values were calculated based on a subsample of 8000 sequences from each dataset. *P < 0.05, **P < 0.01 by two-tailed *t-*test. (**A**) The numbers of observed OTUs increased in both PM and PI. (**B**) The estimated OTU numbers (Chao1) of PM and PI were significantly greater than that of HC. (**C**) Microbial community diversity analysis (Shannon index) showed that the PI microbial community exhibited the greatest diversity. (**D**) Phylogenetic diversity (PD) measures of community diversity.

The variation in overall bacterial community composition based on weighted UniFrac distance measurements was compared among the three groups (Fig. 1B). The variations of the microbial characteristics were similar for the PI sites, but greater for among healthy implants; the difference was statistically significant. Microbial diversity within each sample (the alpha diversity) was calculated at given numbers of reads (n = 8000). Microbial diversity differed significantly between healthy implants and infected sites: 1) OTU richness was higher in plaque samples from peri-implant diseases, compared with samples from healthy implant sites (observed OTUs and Chao 1 index values, Fig. 2A,B); 2) The microbial diversity estimator (the Shannon diversity index) showed that PI sites harbored statistically significantly more diverse bacterial communities than did healthy sites (Fig. 2C); and, 3) the phylogenetic diversity (PD) measure also revealed that the microbial communities of PI sites were the most diverse, and those of healthy implant sites were the least diverse (Fig. 2D).

### Healthy implant sites and peri-implantitis sites harbor distinct bacterial communities

**Figure 3:**
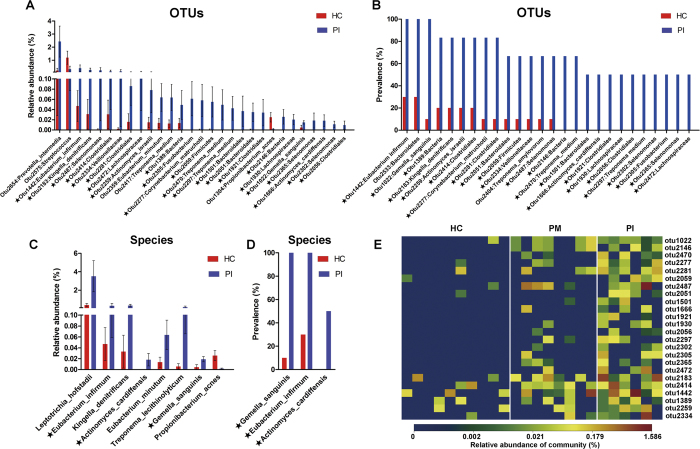
OTUs and taxa differing between healthy implant (HC) and peri-implantitis (PI) sites. (**A**) A total of 29 OTUs exhibited significant differences in mean relative abundances between HC and PI sites (Wilcoxon rank-sum test, P < 0.05). The bars show mean ± SEM relative abundances. In total, levels of 27 OTUs were higher in PI. (**B**) OTUs differing in terms of detection frequency between HC and PI sites (Fisher’s exact test, P < 0.05). (**C**) Species differing in terms of relative abundance between HC and PI sites (Wilcoxon rank-sum test, P < 0.05). The bars show mean ± SEM relative abundances. (**D**) Species differing in terms of detection frequency between HC and PI sites (Fisher’s exact test, P < 0.05). OTUs or species marked with stars (★) differed significantly in terms of both relative abundance and detection frequency. (**E**) A heat map of the relative abundances of OTUs that differed significantly between health and disease. The diagram shows OTUs that differed both in relative abundance (Wilcoxon rank-sum test, P < 0.05) and frequency of detection (Fisher’s exact test, P < 0.05) in HC and PI sites. Peri-implant mucositis sites were intermediate in terms of both relative abundance and prevalence.

**Figure 4:**
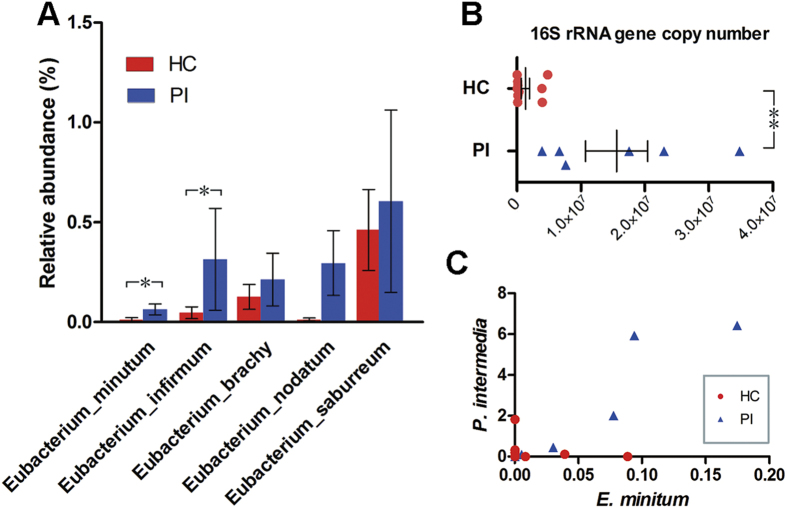
Members of the genus *Eubacterium* in healthy implant (HC) and peri-implantitis (PI) sites. (**A**) The relative abundances of *Eubacterium* species were compared. Bars represent the means ± SEMs of the relative abundances of detected species. *P < 0.05 by the Wilcoxon rank-sum test. **(B)** Total abundances, measured via real-time qPCR, of the *Eubacterium brachy* subgroup (including *E. brachy*, *E. infirmum*, *E. nodatum*, and *E. tardum*). **P < 0.01 by Wilcoxon rank-sum test. (**C**) Positive correlation between *Eubacterium minutum* and *Prevotella intermedia*.

Analysis of the relative abundance of microbial taxonomic groups showed that bacterial compositions differed between healthy and PI sites. Generally, the dominant phyla at implant sites were Firmicutes, Bacteroidetes, Fusobacteria, Actinobacteria, and Proteobacteria. Other dominant taxa are described in Figure S1-S2. The relative abundance levels of 29 OTUs differed significantly between healthy implants and PI sites, of which 27 were over-represented in the latter sites (P < 0.05, Wilcoxon rank-sum test; Fig. 3A). A total of 26 OTUs showed higher prevalence in PI sites than in healthy implants (P < 0.05, Fisher’s exact test; Fig. 3B). A total of 24 OTUs showed higher relative abundance and prevalence in PI sites compared with in healthy implants. Analysis at the species level showed that the relative abundances of *Leptotrichia hofstadii*, *Eubacterium infirmum*, *Kingella denitrificans*, *Actinomyces cardiffensis, Eubacterium minutum*, *Treponema lecithinolyticum,* and *Gemella sanguinis* were higher in PI sites, whereas *Propionibacterium acnes* showed lower proportion. *Gemella sanguinis*, *Eubacterium minutum,* and *Actinomyces cardiffensis* were more prevalent in PI sites (Fig. 3C,D). The proportions of other members of *Eubacterium*, including *Eubacterium brachy, Eubacterium nodatum,* and *Eubacterium saburreum*, were also higher, although the differences were not significant (Fig. 4A). Real-time PCR using genus-specific primers showed that the bacterial load of the *Eubacterium brachy* subgroup (as measured by 16S rRNA gene copy number) was significantly higher in PI sites compared with healthy implants. The real-time PCR results were in agreement with OTU-based analysis, confirming that members of *Eubacterium* were more abundant in PI sites (Fig. 4B). Analysis of the co-occurrence revealed a positive correlation between *Eubacterium minutum* and *Prevotella intermedia*, a periodontal pathogen^27^ (Fig. 4C).

### The microbial communities of PM sites were intermediate in nature between those of healthy implants and PI sites

From a microbial viewpoint, PM appears to be a transitional phase on the course to PI. It is likely that the microbial characteristics of PM are intermediate between those of healthy implants and PI site. 1) The extent of variation in the microbial community of PM was intermediate between that of healthy implants and PI site (Fig. 1B); 2) PM was also intermediate in terms of alpha diversity (Fig. 2). PM was associated with greater bacterial diversity than were healthy implant sites, but lower than that of PI sites; 3) PI-associated OTUs were of moderate relative abundance (Fig. 3E).

**Figure 5:**
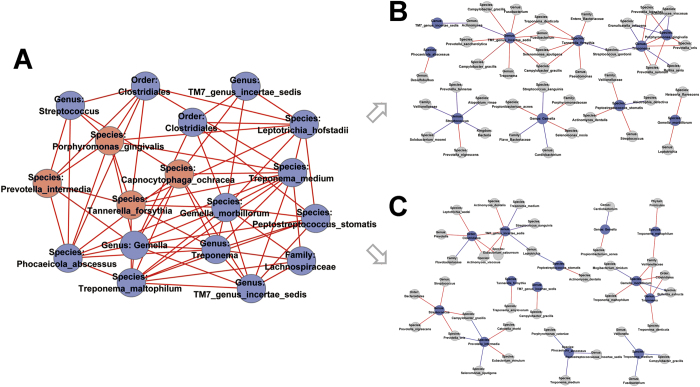
Co-occurring network modules in PM site and corresponding OTUs in HC and PI sites. Edges between each pair of OTUs indicate significant correlations (P < 0.01 by permutation test). Red and blue edges indicate positive and negative correlations, respectively. (**A**) Module in PM network consisted of OTUs with at least five degrees. Periodontal pathogens were marked red. (**B, C**) Corresponding OTUs did not cluster into pairwise modules in HC and PI sites.

We further analyzed the co-occurrence network of microbiota in peri-implant sites, and the results revealed that the microbial components were strongly connected (Figure S3–S5). Interestingly, we found unique and clearly delimited modules for PM with18 nodes (Fig. 5A). Modules of PM networks consisted of nodes with at least five degrees. Periodontal pathogens, such as *Porphyromonas gingivalis*, *Tannerella forsythia*, *Prevotella intermedia* and *Capnocytophaga ochracea* were clustered as part of the module. However, corresponding nodes did not form any pairwise modules in HC and PI sites (Fig. 5B,C).

### The core subgingival microbiome of healthy implant and peri-implant diseases

**Figure 6:**
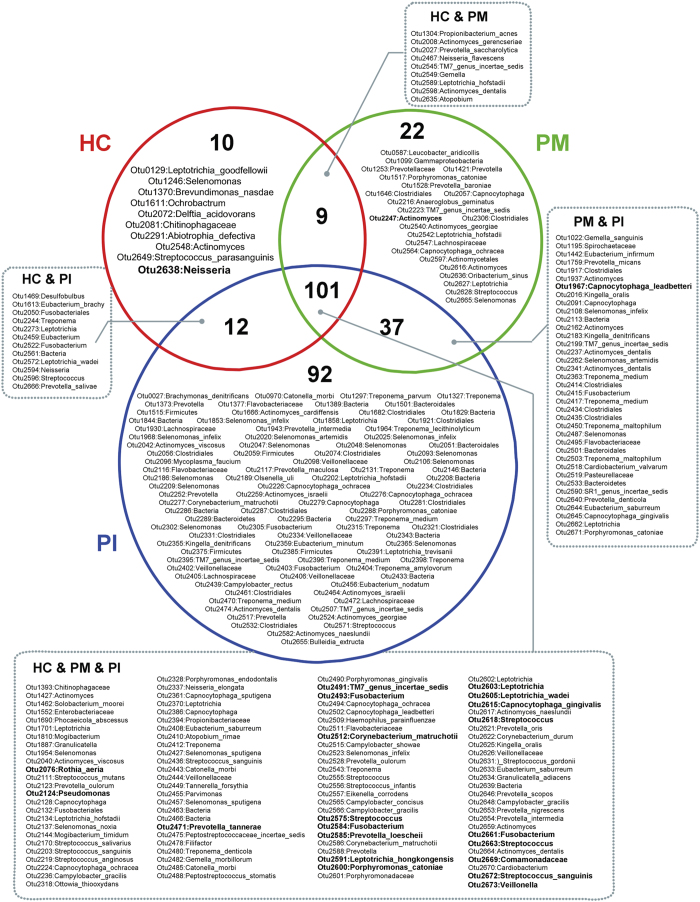
Venn diagram of the core microbiome of peri-implant sites. Each circle (red, green or blue) contains OTUs present in at least 50% of subjects within a group. OTUs in the overlapping regions were shared by two or three groups. Numerically dominant OTUs with mean relative abundances >0.5% are shown in bold.

Several taxa differed between healthy implant and PM sites (Figure S6) and between PM and PI sites (Figure S7). We defined the core peri-implant microbiome. Generally, healthy implant, PM, and PI sites shared most OTUs. Of the 383 OTUs present in at least 50% of all subjects, 101 were common to all subjects; these included members of *Streptococcus*, *Leptotrichia*, *Actinomyces*, *Capnocytophaga*, *Prevotella*, *Fusobacterium*, *Neisseria,* and TM7. These taxa predominated in all subjects (Fig. 6). Healthy implant sites shared few OTUs with PM or PI. Ten OTUs were more prevalent in healthy implant sites, suggesting associations between such bacteria and implant health. PM and PI sites shared 37 OTUs, and 151 OTUs were more prevalent in peri-implant diseases. In total, 22 OTUs were unique to PM sites and 92 to PI sites.

## Discussion

We have described the complexity of microbial communities in peri-implant sites, and we found that peri-implant diseases were associated with dysbiotic subgingival microbial communities. Our study indicates the important role played by the microbiota in peri-implant diseases.

We measured the complexity of microbial communities in PI sites, which contained larger numbers of OTUs and had higher Shannon index values than did healthy implant sites. These results were confirmed by showing that the presence of non-abundant OTUs explained the increase in microbial diversity. The results are consistent with previous findings of microbial enrichment in ailing implant sites^23^,^28^,^29^. However, our results contradict some earlier report, which claimed that PI was attributable to a simple infection of relatively low microbial diversity^16^. This may be explained by differences in sampling methods (a periodontal probe vs. a pointed piece of paper). The paper point sampling method may collect only the superficial region of a submucosal biofilm^30^, thus underestimating the richness and diversity of the microbial community around a dental implant. In our present study, we obtained plaque from the deepest pockets of PI sites using periodontal probe. To allow results to be comparable, healthy implants, which lack deep peri-implant pockets, were also sampled from shallow peri-implant sulci using periodontal probe instead of curette. In recent work using the 16S rRNA gene pyrosequencing method^31^, no significant difference in the microbial diversity of healthy and ailing implants was found (although the Shannon index of ailing implants was higher), which is in part explained by the fact that PM and PI patients were both assigned to the diseased group. In summary, our results indicate that peri-implant diseases are associated with changes in the microbial enrichment (healthy implant < peri-implant mucositis < peri-implantitis). Implants and teeth share histopathological and ecological similarities, and it has thus been proposed that the microbial communities around these structures should be similar^6^,^22^,^32^,^33^,^34^. Evidence that the host responses to microbiota differ at implants and teeth is lacking. However, recent studies have shown that microbial peri-implant communities differ markedly from those of periodontal sites^16^,^31^; the former sites exhibited lower microbial diversity and a simpler microbial composition. In the present study, we did not seek to address the similarity (or otherwise) of the microbiota of teeth and dental implants. However, the dominant bacterial taxa in the plaque in peri-implant sites were similar to those of teeth (Figure S2)^35^,^36^. We assumed that progression of a healthy implant to PI was similar to the development of periodontitis, and, in this preliminary study, we aimed to identify potential “key pathogens” of peri-implant diseases, which may be less abundant in health. At the present level of sequencing depth, we found that the most significant difference between PI and healthy implant sites was associated with the levels of poorly abundant OTUs, or taxonomic “species.”

Periodontal pathogens including *Porphyromonas gingivalis, Treponema denticola, Tannerella forsythia, Aggregatibacter actinomycetemcomitans, Prevotella intermedia, Fusobacterium*, and *Campylobacter* have been reported to be associated with implant diseases^22^,^37^,^38^,^39^. The relative abundances of *Treponema denticola, Prevotella intermedia, Fusobacterium*, and *Campylobacter *were markedly increased in the lesion sites in our study, but the differences were not significant upon Wilcoxon rank-sum testing (data not shown). The relative abundances of *Porphyromonas gingivalis* and *Tannerella forsythia* were similar in healthy implant and PI sites, but were reduced in PM site (data not shown). Taken together, our data were largely consistent with those of previous studies^23^,^40^. Moreover, analysis at the OTU level identified several suspected periodontal pathogens, which increased in relative abundance, and prevalence, in PI sites. Such suspects included members of *Eubacterium, Treponema*, and *Selenomonas*. Particularly, *Eubacterium *spp. appear to be promising candidate peri-implant pathogens. Our results agree with those of previous reports that *Eubacterium* spp. were of higher relative abundance, and present in greater numbers, at peri-implantitis sites^41^. Also, *Eubacterium* species such as *Eubacterium minutum* and *Eubacterium nodatum*, the numbers of which were increased significantly at PI sites, have been associated with periodontitis^36^,^42^,^43^. Moreover, *Eubacterium minutum* were likely to co-exist with *Prevotella intermedia*, a well-known periodontal pathogen^27^. Our study highlights the urgency of conducting further research on the role played by Eubacterium in peri-implant diseases.

Although lacking sufficiently large samples, we used network analysis to clarify the co-occurrence patterns of microbial communities in peri-implant sites. In this study, microbial networks were constructed based on the inter-taxa correlations of closely related bacteria co-existing in subgingival sites of implants. Networks of co-occurring microbial taxa in implant sites consisted mainly of periodontal bacteria, implying a niche similarity between periodontal and peri-implant sites^36^. Positively correlated microbial taxa may have interactions, such as habitat affinities and symbiotic relationships^44^. As random associations between the taxa in the networks are expected, a module network was constructed using strongly linked taxa that may play core roles in the interactions with other taxa. We found that periodontal pathogens such as *Porphyromonas gingivalis*, *Tannerella forsythia*, and *Prevotella intermedia* were among members of the module in the PM co-occurring network, indicative of their potential biotic interaction with other microbes during the early stages of peri-implant diseases. The “keystone pathogen” hypothesis suggests that pathogens of low abundance can cause inflammatory diseases by rendering a symbiotic microbial community dysbiotic^45^. Based on network analysis, it is possible that periodontal pathogens play key roles and contribute to an overall shift in the microbial community, despite their low abundance.

We defined the core microbiome of healthy peri-implant, PM, and PI sites, defined as the “most commonly detected OTUs across samples”. Overall, we found that the OTUs were similar in the three groups. Most OTUs were found in all individuals, indicating that microbial ecosystems of peri-implant sites are similar. Certain OTUs—such as the *Streptococcus, Leptotrichia, Capnocytophaga, Prevotella, Fusobacterium, Neisseria,* and *Rothia* genera—were dominant in all samples. We also aimed to describe the core microbiota associated with health and disease. For example, OTUs identified as *Neisseria spp.* were found in most healthy implant samples, and at relatively high abundances (>0.5%). Although this taxon remains unclassified at the species level, these bacteria may maintain health. Many OTUs present in the majority of PI samples were rare in the other two groups. Such PI-associated OTUs contributed to the complexity of the submucosal microbial community.

We also investigated the microbial diversity in PM sites, the clinical parameters of which were intermediate between those of healthy implants and PI. These sites appeared to not exhibit a distinct microbial pattern, rather hosting candidate pathogens causing PI. This reflects the fact that, from a microbiological viewpoint, PM precedes PI, and should be considered an early event in the development of PI. Our results are in accordance with the consensus of the Seventh European Workshop on Periodontology^46^. We acknowledge, however, that we cannot presently conclude that PM definitely progresses to PI, although inflammation generally develops rapidly around dental implants^47^. There is a causal relationship between gingivitis and periodontitis, but further work is needed to evaluate the relationship between PM and PI^48^. Periodontal pathogens such as *Porphyromonas gingivalis*, *Tannerella forsythia*, *Prevotella intermedia* and *Capnocytophaga ochracea*, clustered together in PM sites, suggesting that periodontal pathogens may play important roles in the pathogenesis of peri-implant diseases.

In conclusion, we have described and compared the microbial communities of healthy implant, PM, and PI sites, affording deep insight into the dysbiosis in the microbial community of ailing implants. Our work adds to the current knowledge that periodontal pathogens may play important roles in oral peri-implant diseases.

## Additional Information

**How to cite this article**: Zheng, H. *et al.* Subgingival microbiome in patients with healthy and ailing dental implants. *Sci. Rep.* **5**, 10948; doi: 10.1038/srep10948 (2015).

## Supplementary Material

Supplementary Information

## Figures and Tables

**Table 1:** Demographic and clinical characteristics of all subjects.

**Characteristic**	**Healthy subjects (n=10)**	**Peri-implant mucositis patients (n=8)**	**Peri-implantitis patients (n=6)**
Male/female	3/7	6/2	3/3
Age (years ± s.d.)	42.6 ± 3.6	46.0 ± 3.5	48.2 ± 7.8
Years of functional loading (years ± s.d.)	3.9 ± 0.7	4.2 ± 0.7	4.5 ± 1.4
Plaque index (mean ± s.d.)	1.3 ± 0.5	1.4 ± 0.5	1.16 ± 0.4
Peri-implant probing depth (mm ± s.d.)	2.0 ± 0.9	2.0 ± 1.6	4.0 ± 2.7
Bone loss (mm ± s.d.)	0	0	2 ± 3.6
Bleeding on probing (+/–)	0/10	5/3	6/0
