# Supplementary material for: Subgingival microbiome in patients with healthy and ailing dental implants
Source: Sci Rep. 2015 Jun 16;5:10948. doi: 10.1038/srep10948 (PMC4468443; doi:10.1038/srep10948)
Supplement: Supplementary Information [file srep10948-s1.pdf]

Subgingival microbiome in patients with healthy and ailing dental implants

Hui Zheng[1*], Lixin Xu[2*], Zicheng Wang[3], Lianshuo Li[3], Jieni Zhang[1], Qian Zhang[4], Ting Chen[5,6#], Jiuxiang Lin[1#] & Feng Chen[4#]

[1] Department of Orthodontics, Peking University School and Hospital of Stomatology, Beijing 100081, China, [2] The Third Dental Center, Peking University School and Hospital of Stomatology, Beijing 100011, China, [3] Bioinformatics Division, TNLIST and Department of Automation, Tsinghua University, Beijing 100084, China, [4] Central Laboratory, Peking University School and Hospital of Stomatology, Beijing 100081, China, [5] Bioinformatics Division, TNLIST and Department of Computer Science and Technology, Tsinghua University, Beijing 100084, China, [6] Program in Computational Biology and Bioinformatics, University of Southern California, Los Angeles, CA 90089, USA.

[*] These authors contributed equally to this work.

# Correspondence and requests for materials should be addressed to:

Feng Chen: 22 Zhongguancun Nandajie, Haidian District, Beijing 100081, China. Tel: +86-010-82195773. Fax: +86-010-82195773. Email: moleculecf@gmail.com;

or

Jiuxiang Lin: 22 Zhongguancun Nandajie, Haidian District, Beijing 100081, China. Tel: +86-010-82195269. Fax: +86-010-82195336. Email: jxlin@pku.edu.cn;

or

Ting Chen: FIT 1-107, Tsinghua University, Beijing, 100084, China. Tel: +86-010-62797101.

Email: tingchen@mail.tsinghua.edu.cn.

Supplementary Methods

Subject recruitment

Ten individuals with healthy peri-implant sites (n = 10), eight cases with PM (n = 8), and six cases with PI (n = 6), participated in the study. All subjects were medically healthy; did not suffer from any systemic illness; were not pregnant; did not have diabetes; and had not taken antibiotics, anticoagulants, or non-steroidal anti-inflammatory drugs in the 6 months prior to the study. All were non-smokers. All subjects were partially edentulous patients due to severe periodontitis. They had received initial periodontal therapy (scaling and root planning) and periodontal surgery (if required). All patients commenced well-supervised maintenance care (supportive periodontal therapy) before implant treatment. Straumann Dental Implant System (Straumann, California, USA) was used, and the implants functioned for at least 1 year after the prosthesis were adopted. The project was approved by the Peking University Biomedical Ethics Committee (Beijing, China). Subjects gave written informed consent with the approval of the Ethics Committee of the Peking University School and Hospital of Stomatology.

Diagnosis and sample collection

Oral examination and diagnosis were performed by one dentist, using visual, probing, and radiographic methods. Intra-oral periapical radiographs were obtained using the parallel technique. Dogora imaging software was used for analysis of peri-implant bone loss by the same examiner. Average bone level on the mesial and distal aspect of each implant was accessed, using the implant-abutment junction as the reference point. Dimensional distortions and enlargements on the radiographs were adjusted. The diagnostic criteria for peri-implant diseases were in accordance with the recognized definitions of PM and PI [1]. In brief, peri-implant tissue that did not bleed on probing, which was not suppurating, and for which radiography yielded no evidence of marginal bone loss, was classified as healthy. PM was diagnosed when an implant showed clinical signs of inflammation but no evidence of bone loss. PI was diagnosed based on loss of marginal bone in conjunction with inflammation of the peri-implant mucosa, as evidenced by bleeding and/or

suppuration after probing. The clinical signs of inflammation for this study include bleeding on probing, increased probing depths, mucosal swelling/hyperplasia and mucosal recession [2]. Plaque samples were collected from peri-implant sulci or pockets, at the maximum possible probing depth, using a sterile metal periodontal probe. Samples were suspended in 1-mL sterile tubes containing 200-μL amounts of TE buffer (20 mM Tris, 2 mM EDTA; pH = 7.4) and frozen at -80°C prior to DNA isolation.

Microbial DNA extraction, 16S rRNA gene library preparation, and pyrosequencing

DNA from plaque samples was extracted using a TIANamp Bacteria DNA Kit (Tiangen Biotech, Beijing, China), following the manufacturer's instructions after initial treatment with lysozyme (20 mg/mL, 37°C for 1 h). DNA concentrations were measured using a Qubit Fluorometer (Invitrogen, California, USA) and via qPCR. The amount of DNA per sample was 0.24-1.62 μg.

The v1-v3 hypervariable regions of bacterial 16S ribosomal RNA genes were amplified via PCR. The PCR primers were 27f: 5′-AGAGTTTGATCCTGGCTCAG-3′ [3], and 534r: 5'-ATTACCGCGGCTGCTGG-3' [4], with 10-nt barcodes tagged to the 5′-ends. PCR was performed as described in the manual of the GS FLX Amplicon DNA library preparation method (Roche, Mannheim, Germany). Briefly, genomic DNAs were used as templates. Cycling involved initial denaturation at 94°C for 3 min; 30 cycles of 94°C for 30 s, 57°C for 45 s, and 72°C for 60 s, followed by a final extension at 72°C for 2 min. The libraries were pyrosequenced on a 454-GS-FLX sequencing platform (454 Life Sciences, Branford, USA) at the BGI Institute (BGI Institute, Shenzhen, China).

16S data processing and statistical analysis

In total, 24 samples were sequenced, and the raw data (*.sff files) generated were analyzed using (principally) the pipeline tools MOTHUR [5] and QIIME [6]. In brief, sequences were demultiplexed based on a unique barcode assigned to each sample. To filter low-quality sequences, those with average quality scores ≤25 and sequence lengths <200 nt were

discarded. A maximum of one barcode correction was allowed at this stage, no primer mismatch, 6 ambiguous bases were permitted. Trimmed reads were clustered into operational taxonomic units (OTUs) at a 97% similarity cutoff using the *de novo* OTU selection strategy. Taxonomies were assigned by the RDP classifier (version 1.27), with a confidence threshold of 0.8 [7] (**Figure S1**). After we obtained OTU tables and phylogenetic trees, microbial richness estimators (Observed OTUs, Chao1), evenness estimators (Equitability), diversity estimators (Shannon Index, Simpson Index), and phylogenetic distances (PDs), were calculated using Perl scripts. Fixed numbers of sequences were randomly selected from each dataset to generate rarefaction curves and allow microbial diversity to be estimated. Weighted UniFrac distances were estimated within and between groups, based on the OTU tables and the phylogenetic trees [8]. Relative abundances of microbial taxa at each of the phylum, class, order, family, genus, and species levels were calculated and compared. The unpaired student's *t*-test was used to compare alpha and beta diversities. Differences in the relative abundances of taxa in healthy implant, PM, and PI samples were analyzed using the Wilcoxon rank-sum test. Differences in prevalence were compared using Fisher's exact test. P values < 0.05 were considered to indicate statistical significance. For each group of samples, OTUs observed in at least half of the samples were used to construct an OTU network [9,10]. We calculated the Pearson correlation coefficients (PCC) for each pair of OTUs and used the permutation test to compute the statistical significance of the PCC value. Edges were set between pairs of OTUs for which the PCC was significant ($P<0.01$).

Quantification of bacterial loads of the Eubacterium brachy subgroup

Bacterial loads of members of the *Eubacterium brachy* subgroup were determined via real-time PCR using modified genus-specific primers (Forward: 5'-ACACGGTCCAAACTCCTACG-3', Reverse: 5'-TTCGCRTCCCAAATTCCG-3') [11]. First, 16S rRNA genes were amplified using universal bacterial primers (27f/1492r) and the PCR products purified with the aid of a TIANquick Midi Purification Kit (Tiangen Biotech, Beijing, China). The DNA levels were adjusted to 10 ng/μL; these solutions served as templates. Each PCR reaction

was performed in a volume of 20 μL, containing 10 μL Power SYBR Green PCR Master Mix (Applied Biosystems, Warrington, UK), 75 nM primers, and 1 μL (10 ng) DNA template. The qPCR cycling conditions were 95°C for 2 min; followed by 40 cycles of 95°C for 15 s and 60°C for 1 min. PCR amplicons of the *Eubacterium brachy* subgroup served as standards (500 pg/μL, 50 pg/μL, 5 pg/μL, 500 fg/μL, and 50 fg/μL). The presence and specificity of qPCR products were evaluated by melting curve analysis and agarose gel electrophoresis. All samples and standards were amplified in triplicate, and mean values were used in the analysis. Student's *t*-test was used to determine the significance of differences.

References

1 Zitzmann, N. U. & Berglundh, T. Definition and prevalence of peri-implant diseases. *J Clin Periodontol* **35**, 286-291, doi:10.1111/j.1600-051X.2008.01274.x (2008).

2 Heitz-Mayfield, L. J. Peri-implant diseases: diagnosis and risk indicators. *J Clin Periodontol* **35**, 292-304, doi:10.1111/j.1600-051X.2008.01275.x (2008).

3 Lane, D. J. *et al.* Rapid determination of 16S ribosomal RNA sequences for phylogenetic analyses. *Proc Natl Acad Sci U S A* **82**, 6955-6959 (1985).

4 Muyzer, G., de Waal, E. C. & Uitterlinden, A. G. Profiling of complex microbial populations by denaturing gradient gel electrophoresis analysis of polymerase chain reaction-amplified genes coding for 16S rRNA. *Appl Environ Microbiol* **59**, 695-700 (1993).

5 Schloss, P. D. *et al.* Introducing mothur: open-source, platform-independent, community-supported software for describing and comparing microbial communities. *Appl Environ Microbiol* **75**, 7537-7541, doi:10.1128/AEM.01541-09 (2009).

6 Caporaso, J. G. *et al.* QIIME allows analysis of high-throughput community sequencing data. *Nat Methods* **7**, 335-336, doi:10.1038/nmeth.f.303 (2010).

7 Wang, Q., Garrity, G. M., Tiedje, J. M. & Cole, J. R. Naive Bayesian classifier for rapid assignment of rRNA sequences into the new bacterial taxonomy. *Appl Environ Microbiol* **73**, 5261-5267, doi:10.1128/AEM.00062-07 (2007).

8 Lozupone, C. & Knight, R. UniFrac: a new phylogenetic method for comparing microbial communities. *Appl Environ Microbiol* **71**, 8228-8235, doi:10.1128/AEM.71.12.8228-8235.2005 (2005).

9 Barberan, A., Bates, S. T., Casamayor, E. O. & Fierer, N. Using network analysis to explore co-occurrence patterns in soil microbial communities. *ISME J* **6**, 343-351, doi:10.1038/ismej.2011.119 (2012).

10 Zhang, Z. *et al.* Spatial heterogeneity and co-occurrence patterns of human mucosal-associated intestinal microbiota. *ISME J* **8**, 881-893, doi:10.1038/ismej.2013.185 (2014).

11 Spratt, D. A., Weightman, A. J. & Wade, W. G. Diversity of oral asaccharolytic Eubacterium species in periodontitis--identification of novel phylotypes representing uncultivated taxa. *Oral Microbiol Immunol* **14**, 56-59 (1999).

Supplementary Figures

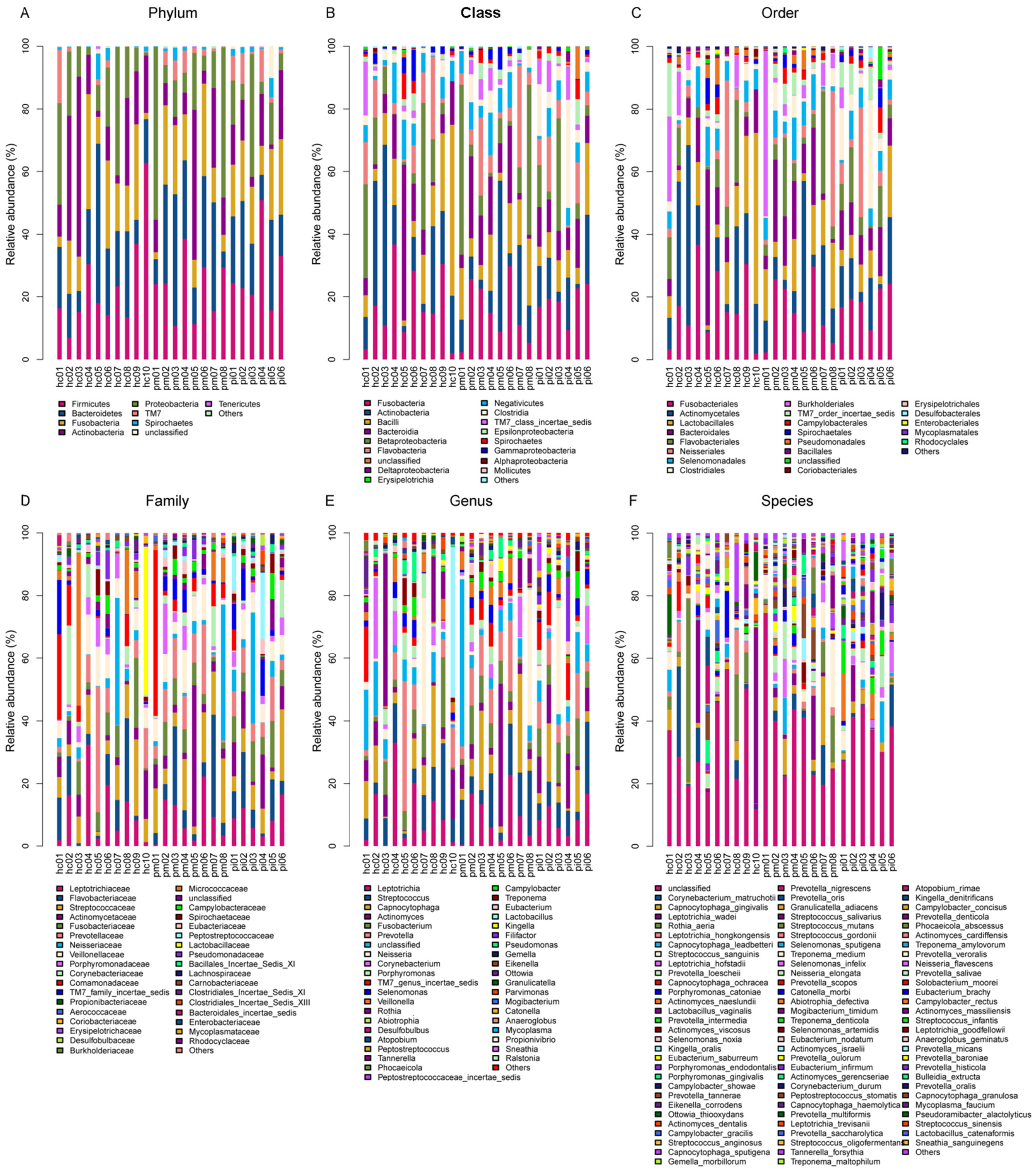

Figure S1. Microbial compositions of all samples at various taxonomic levels. Each column shows the relative abundance of microbial components in a single sample. Sequence annotation was performed with the aid of the Ribosomal Database Project (RDP). The percentages of OTUs successfully assigned to taxonomic levels were: phylum (89.97%), class (87.84%), order (87.24%), genus (71.53%), and species (42.84%).

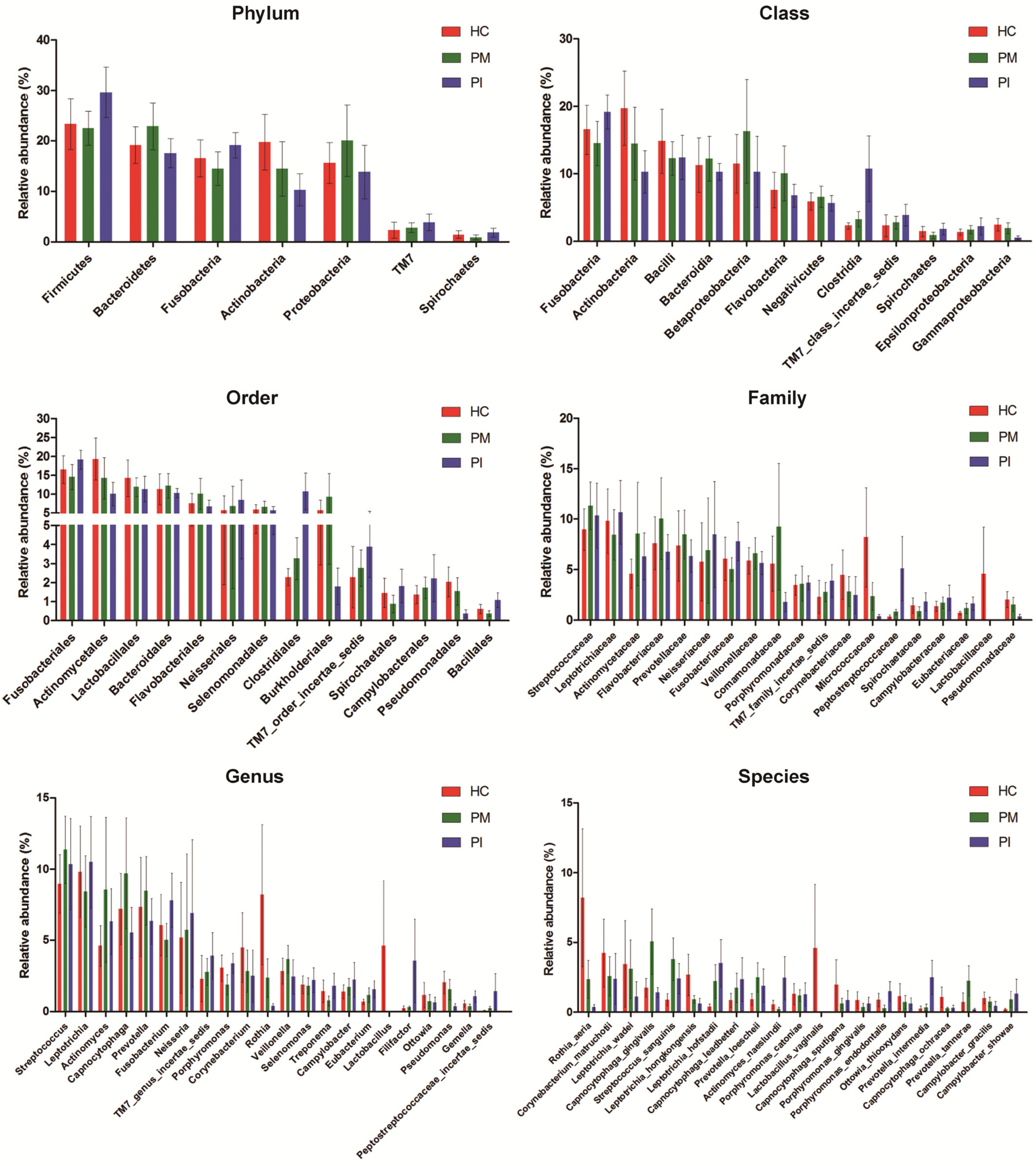

Figure S2. Bacterial taxonomic profiles of healthy implant (HC), peri-implant mucositis (PM), and peri-implantitis (PI) sites. The graphs show the predominant taxa in HC, PM, and PI sites at the taxonomic levels of phylum, class, order, family, genus, and species. Taxa of average abundance >0.5% at each level are shown. Bars represent mean ± SEM relative abundances.

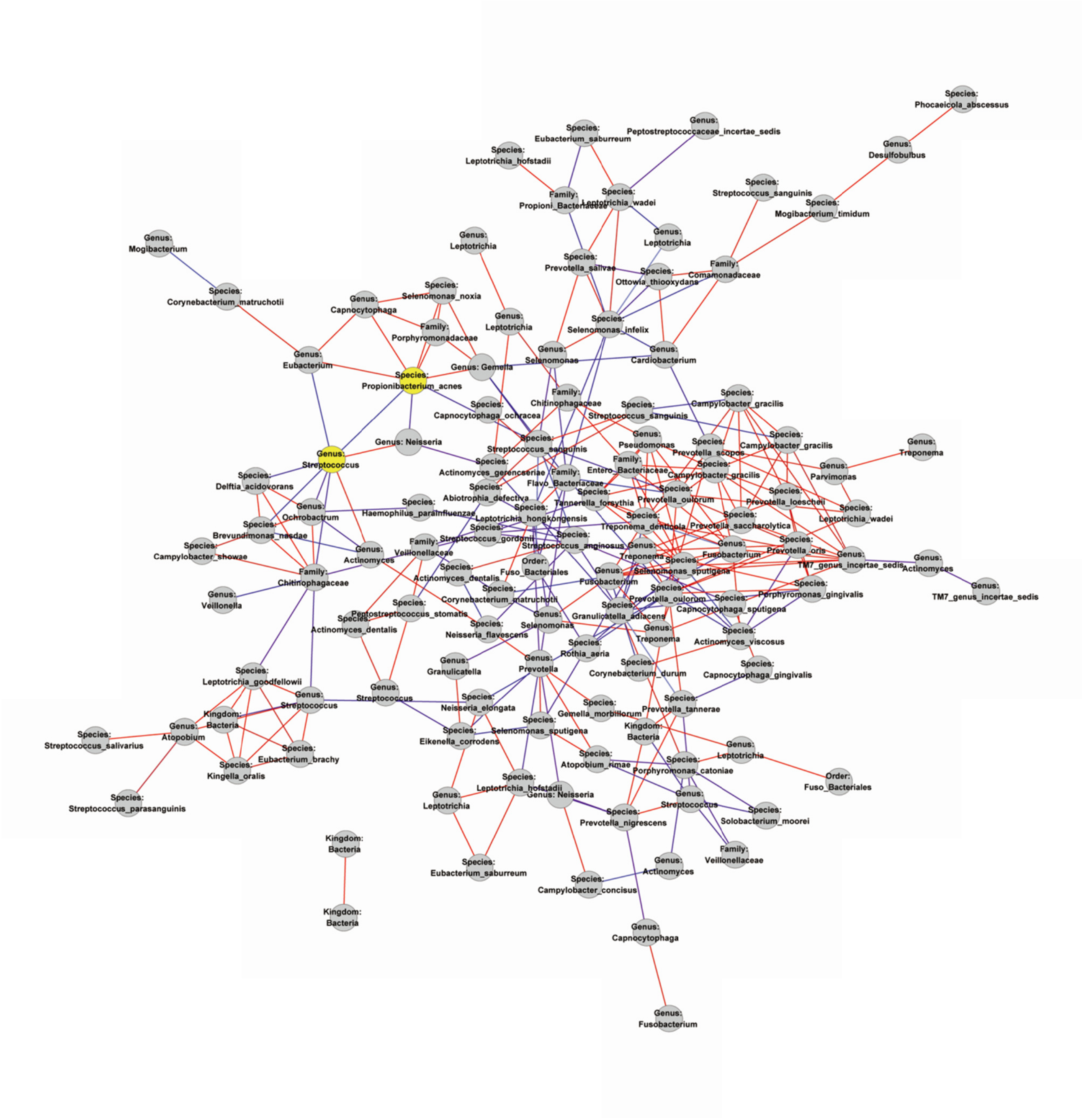

Figure S3. Networks of co-occurring OTUs in healthy implant sites. Edges between each pair of OTUs indicate significant correlations (P<0.01 by permutation test). Red and blue edges indicate positive and negative correlations, respectively. OTUs differing between HC and PI sites (Wilcoxon rank-sum test, P<0.05) are marked in yellow. The microbial co-occurrence network of HC consisted of 120 OTUs with 263 correlations. The hub OTUs (OTUs that have the most linkers) were identified as *Prevotella oulorum*, *Treponema denticola*, *Campylobacter gracilis*, *Selenomonas sputigena*, *Selenomonas infelix*, *TM7 genus incertae sedis* and *Fusobacterium*.

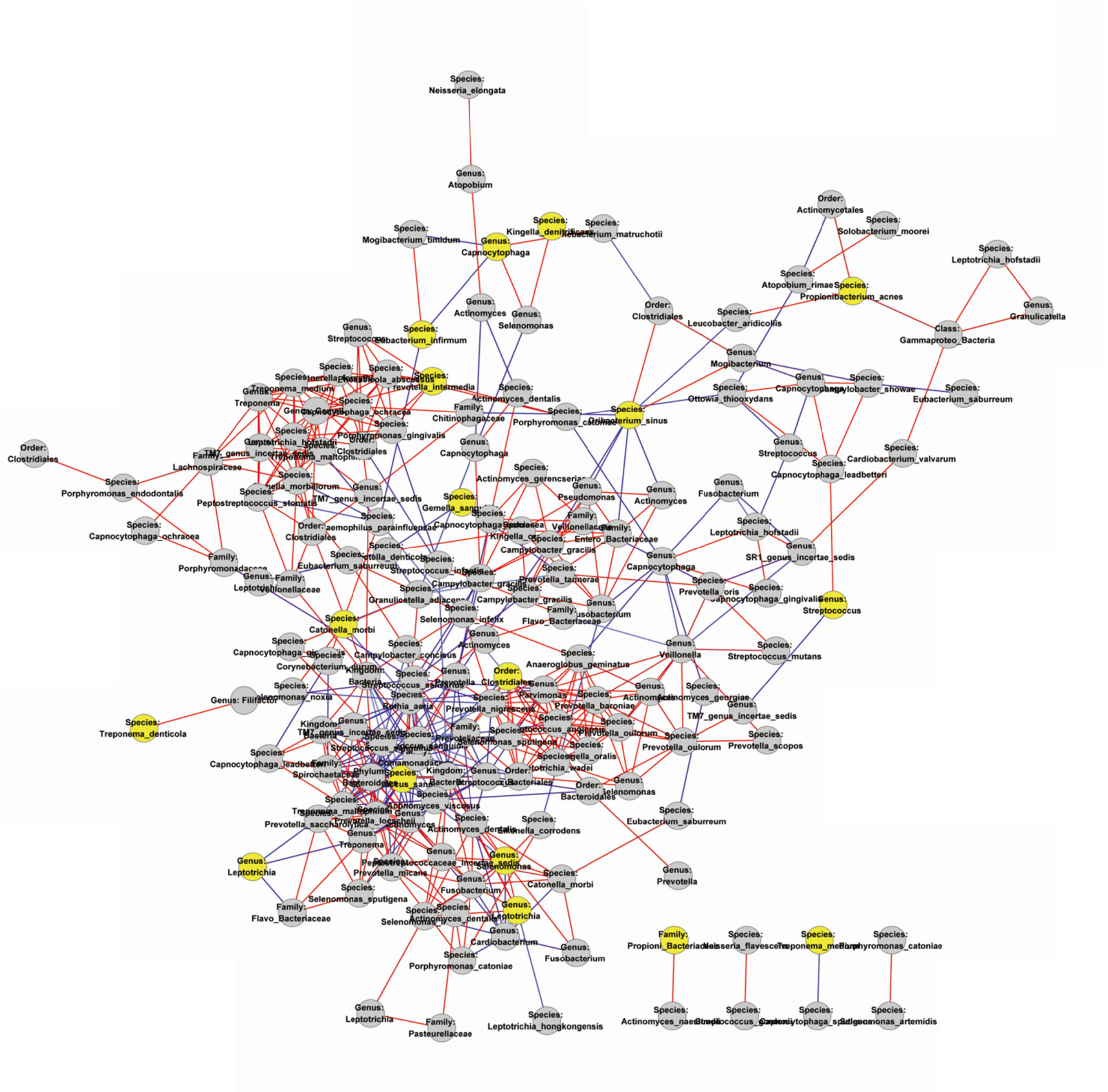

Figure S4. Networks of co-occurring OTUs in peri-implant mocositis sites. Edges between each pair of OTUs indicate significant correlations (P<0.01 by permutation test). Red and blue edges indicate positive and negative correlations, respectively. OTUs differing between HC and PI sites (Wilcoxon rank-sum test, P<0.05) are marked in yellow. A PM network was constructed with 161 OTUs and 520 correlations, in which *Streptococcus sanguinis*, *Rothia aeria*, *Prevotella nigrescens*, *Actinomyces*, *Prevotella loescheii* and *Treponema maltophilum* were the most highly linked taxa.

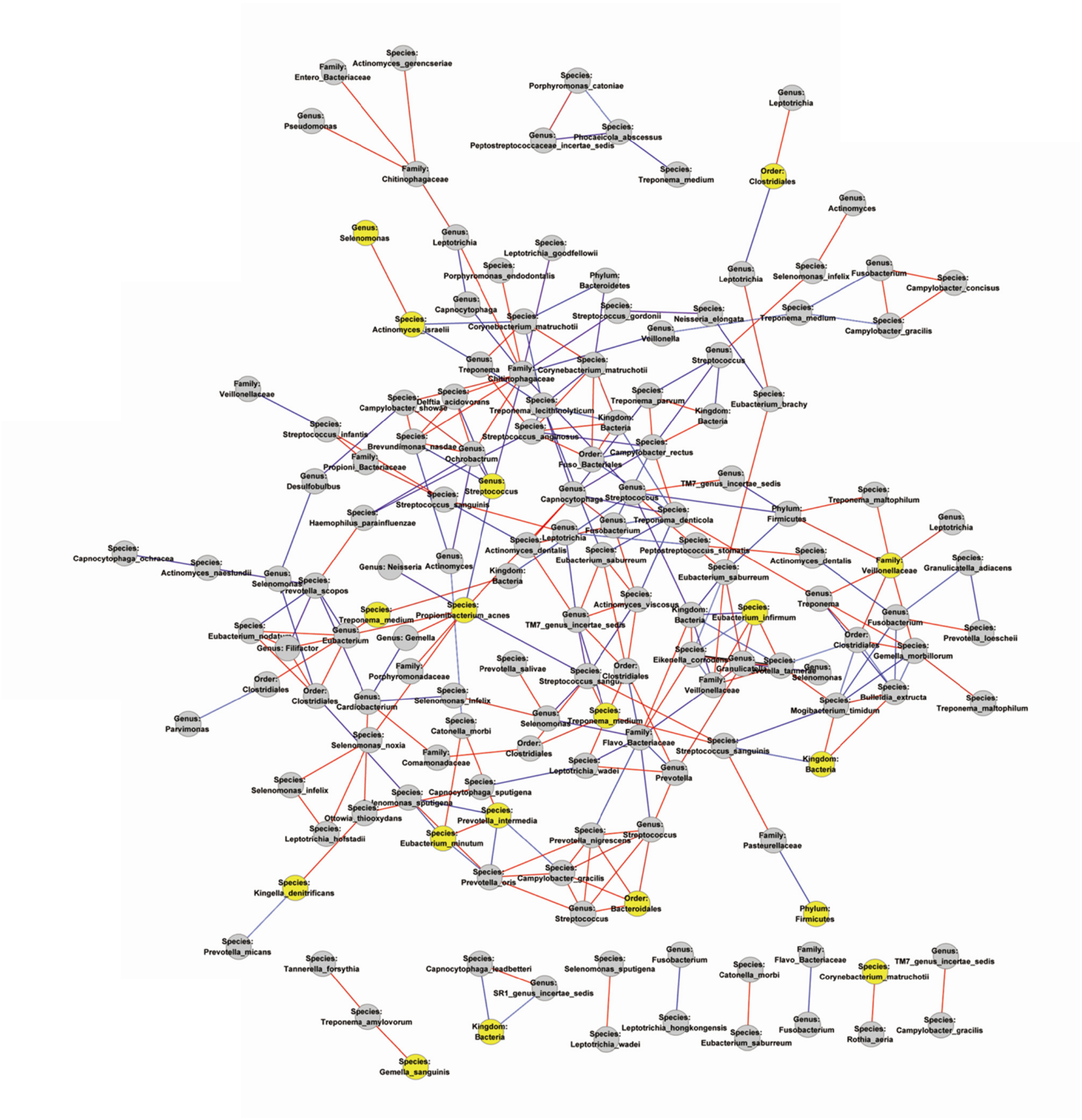

Figure S5. Networks of co-occurring OTUs in peri-implantitis sites. Edges between each pair of OTUs indicate significant correlations (P<0.01 by permutation test). Red and blue edges indicate positive and negative correlations, respectively. OTUs differing between HC and PI sites (Wilcoxon rank-sum test, P<0.05) are marked in yellow. The network consisted of 152 OTUs with 263 correlations and the hub OTUs were *Chitinophagaceae*, *Flavobacteriaceae*, *Propionibacterium acnes*, *Campylobacter rectus* and *Treponema denticola*.

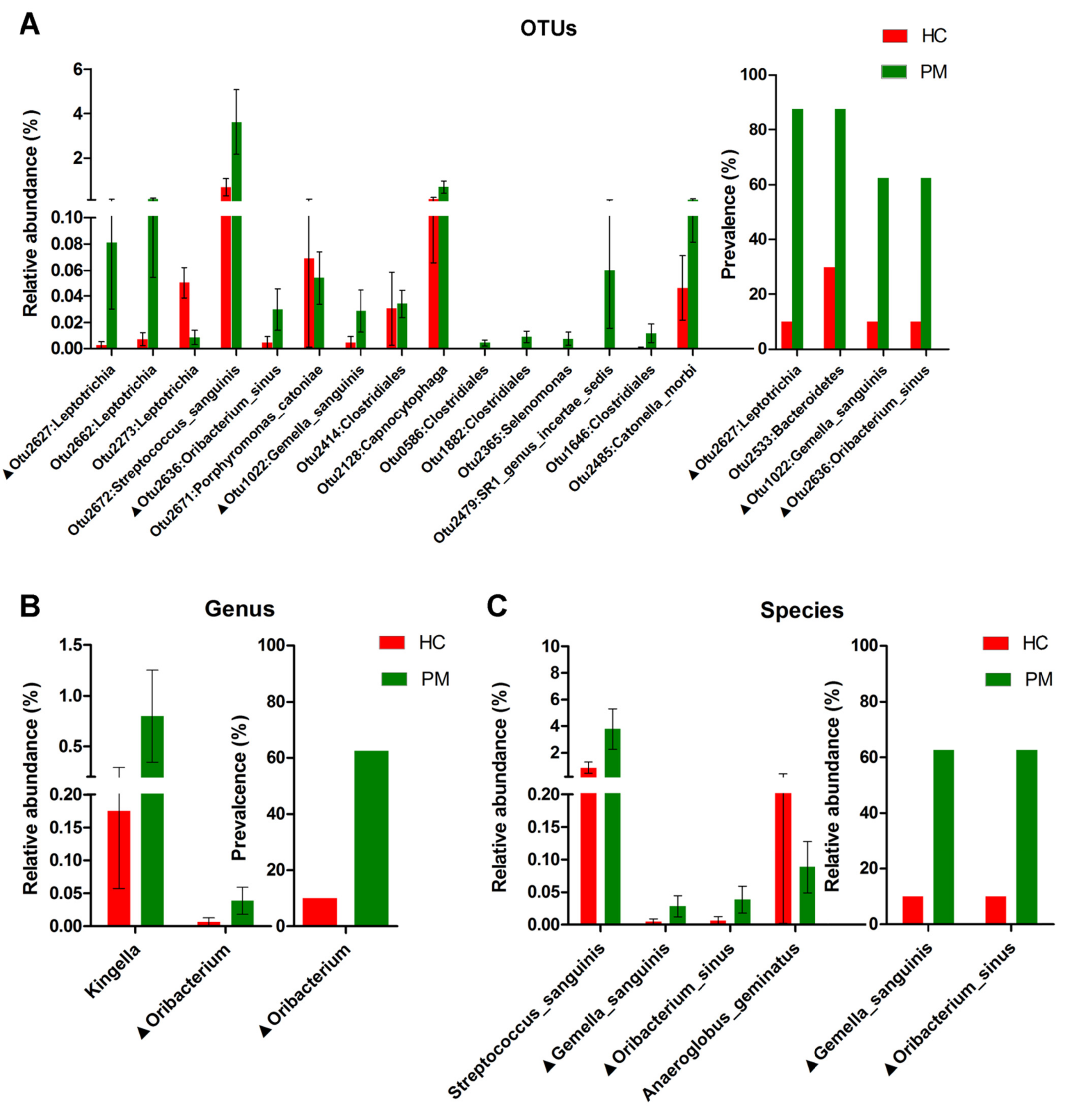

Figure S6. OTUs and taxa differing between healthy implant (HC) and peri-implant mucositis (PM) sites. Differences in relative abundance were analyzed using the Wilcoxon rank-sum test, and prevalences were compared by Fisher's exact test; P<0.05 was considered to reflect a significant difference. The **(A)** OTU level, **(B)** genus level, **(C)** species level. ▲, significant differences in both relative abundance and prevalence. No significant difference was evident at taxonomic levels that are not shown.

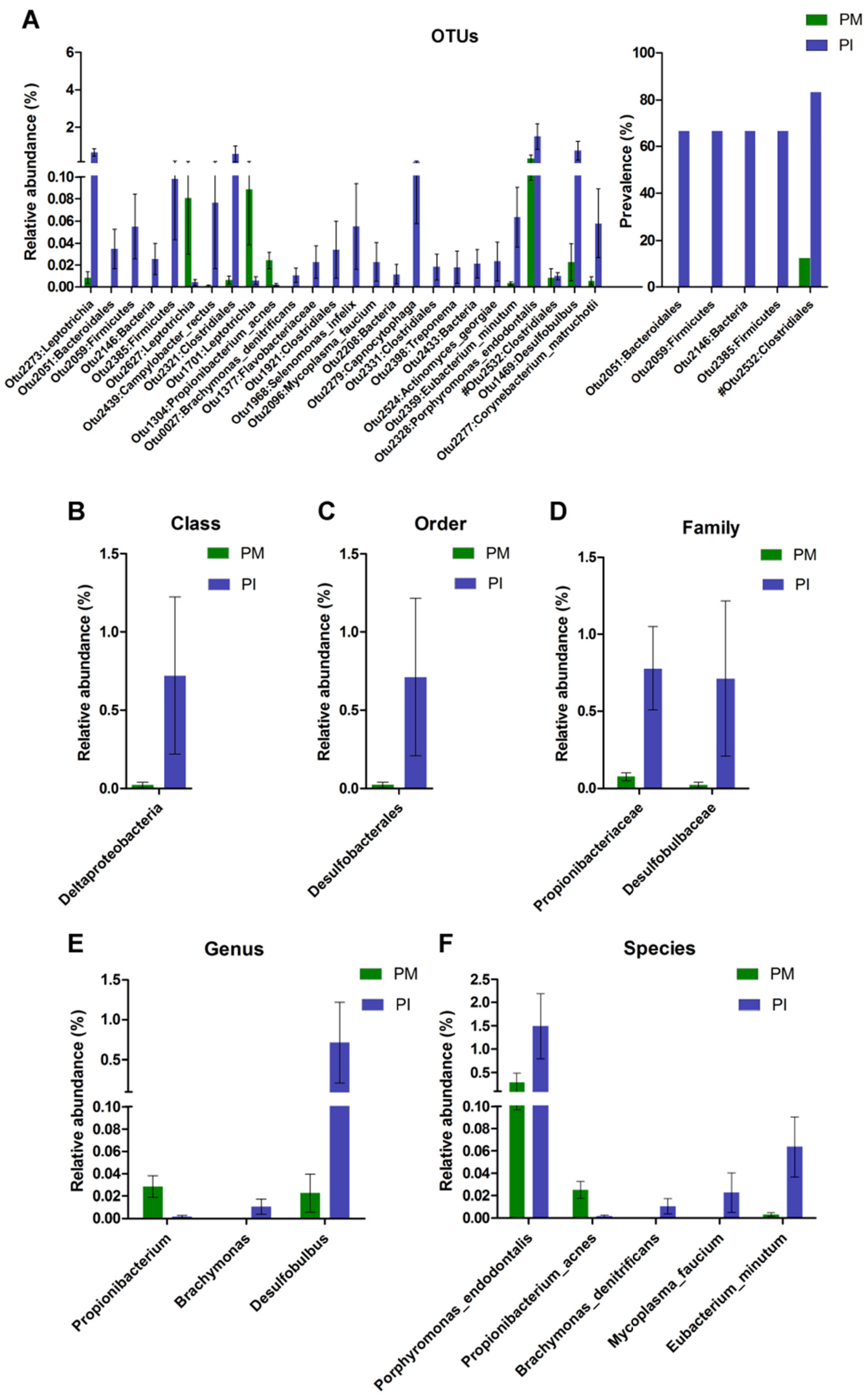

Figure S7. OTUs and taxa differing between peri-implant mucositis (PM) and peri-implantitis (PI) sites. Differences in relative abundance were analyzed using the Wilcoxon rank-sum test, and prevalences were compared by Fisher's exact test; P<0.05 was considered to reflect a significant difference. The **(A)** OTU level, **(B)** class level, **(C)** order level, **(D)** family level, **(E)** genus level, and **(F)** species level. A # sign indicates significant differences in both relative abundance and prevalence. No significant difference was evident at taxonomic levels that are not shown.

Supplementary Tables

Table S1. Summaries of the background information and pyrosequencing data for all samples.

Sample ID	Diagnosis	Gender	Age (years)	Reads after trimming	Number of OTUs
hc01	Healthy	Female	43	15,545	593
hc02	Healthy	Female	43	13,848	542
hc03	Healthy	Female	46	14,095	432
hc04	Healthy	Female	47	13,466	592
hc05	Healthy	Female	43	14,381	573
hc06	Healthy	Male	36	14,964	603
hc07	Healthy	Male	42	16,910	617
hc08	Healthy	Female	44	13,086	311
hc09	Healthy	Female	45	20,525	716
hc10	Healthy	Male	37	15,698	451
pm01	Peri-implant mucositis	Male	49	9,720	421
pm02	Peri-implant mucositis	Male	40	14,478	775
pm03	Peri-implant mucositis	Male	45	13,415	628
pm04	Peri-implant mucositis	Male	46	24,121	789
pm05	Peri-implant mucositis	Female	48	18,678	607
pm06	Peri-implant mucositis	Female	42	21,617	885
pm07	Peri-implant mucositis	Male	50	18,532	650
pm08	Peri-implant mucositis	Male	48	14,442	606
pi01	Peri-implantitis	Male	45	15,751	662
pi02	Peri-implantitis	Female	43	21,768	1,028
pi03	Peri-implantitis	Female	44	23,416	923
pi04	Peri-implantitis	Male	41	10,577	441
pi05	Peri-implantitis	Female	56	38,763	963
pi06	Peri-implantitis	Male	60	26,801	958

Table S2. Microbial diversity estimators for each sample.

Sample ID	Observed OTUs	Chao1	Shannon	Phylogenetic Distance
hc01	593	1016.11	5.61178	40.66184
hc02	542	885.5632	5.196301	37.72005
hc03	432	696.6857	4.139791	28.54832
hc04	592	927.0545	5.690029	36.83339
hc05	573	829.8182	6.34536	36.70331
hc06	603	886.1165	6.176643	40.55881
hc07	617	1059.846	4.825679	39.16441
hc08	311	560.4118	3.843398	23.26342
hc09	716	1098.336	5.592404	42.48262
hc10	451	652.6582	3.944433	28.54155
pm01	421	781.5538	4.327286	26.48408
pm02	775	1393.472	6.894031	47.52957
pm03	628	1042.919	6.095534	41.74441
pm04	789	1274.808	6.759371	47.0232
pm05	607	948.8919	4.678774	39.85641
pm06	885	1447.267	6.341759	53.78853
pm07	650	1086.239	5.366604	37.09711
pm08	606	986.3947	4.819511	37.37356
pi01	662	1037.628	6.052347	45.17445
pi02	1028	1521.014	6.699286	56.45971
pi03	923	1590.5	6.00139	55.18495
pi04	441	664.75	5.746397	28.35377
pi05	963	1364.487	5.759005	58.93781
pi06	958	1597.863	6.362862	57.6682
